# *CsBPC2* is essential for cucumber survival under cold stress

**DOI:** 10.1186/s12870-023-04577-1

**Published:** 2023-11-16

**Authors:** Di Meng, Shuzhen Li, Xiaojie Feng, Qinghua Di, Mengdi Zhou, Xianchang Yu, Chaoxing He, Yan Yan, Jun Wang, Mintao Sun, Yansu Li

**Affiliations:** 1 State Key Laboratory of Vegetable Biobreeding, Institute of Vegetables and Flowers, Chinese Academy of Agricultural Sciences, Beijing, 100081 China; 2 Ganzhou Key Laboratory of Greenhouse Vegetable, College of Life Science, Gannan Normal University, Ganzhou, 341000 China

**Keywords:** Cucumber, Cold stress, *CsBPC2*, Transcriptome sequence

## Abstract

Cold stress affects the growth and development of cucumbers. Whether the *BPC2* transcription factor participates in cold tolerance and its regulatory mechanism in plants have not been reported. Here, we used wild-type (WT) cucumber seedlings and two mutant *Csbpc2* lines as materials. The underlying mechanisms were studied by determining the phenotype, physiological and biochemical indicators, and transcriptome after cold stress. The results showed that *CsBPC2* knockout reduced cucumber cold tolerance by increasing the chilling injury index, relative electrical conductivity and malondialdehyde (MDA) content and decreasing antioxidant enzyme activity. We then conducted RNA sequencing (RNA-seq) to explore transcript-level changes in *Csbpc2* mutants. A large number of differentially expressed genes (1032) were identified and found to be unique in *Csbpc2* mutants. However, only 489 down-regulated genes related to the synthesis and transport of amino acids and vitamins were found to be enriched through GO analysis. Moreover, both RNA-seq and qPT-PCR techniques revealed that *CsBPC2* knockout also decreased the expression of some key cold-responsive genes, such as *CsICE1*, *CsCOR413IM2*, *CsBZR1* and *CsBZR2*. These results strongly suggested that *CsBPC2* knockout not only affected cold function genes but also decreased the levels of some key metabolites under cold stress. In conclusion, this study reveals for the first time that *CsBPC2* is essential for cold tolerance in cucumber and provides a reference for research on the biological function of *BPC2* in other plants.

**Supplementary Information:**

The online version contains supplementary material available at 10.1186/s12870-023-04577-1.

## Introduction

Cucumber (*Cucumis sativus* L.) is an economically important vegetable that is very sensitive to low temperatures [1]. In 2021, the global total production of cucumber was 93,528,796 tons. As the main producing country, China produces 70% of the world’s total yield of cucumber and gherkin [2]. However, long-term low temperatures and short-term critical low temperatures are common problems in winter and spring cultivation in northern China [3]. Low-temperature stress can be divided into cold stress (0–20 °C) and freezing damage (< 0 °C) [4, 5]. Cold stress can mainly change the structure of the cell membrane, reduce the activity of enzymes, deteriorate nutrient absorption capacity, reduce metabolic capacity, etc., which have adverse effects on the normal growth and development of plants [6, 7]. Cucumber can be injured by cold stress during the whole growth and development process, leading to a reduced seed germination rate, yellowing and dying of leaf margins at the seedling stage, a low fertilization rate, a low fruit set rate at the flowering stage, and other issues. In severe cases, the whole plant will die [3], thus affecting the final quality and yield.

Plants have evolved a complex set of cold adaptation mechanisms, including gene transcriptional regulation and a wide range of physiological, biochemical, and metabolic changes [8]. Physiological changes mainly refer to changes in plant cell membranes, protective enzyme systems, osmotic substances, etc. When its levels are increased, malondialdehyde (MDA), which is the end product of membrane lipid peroxidation under cold stress conditions, can cross-link and polymerize with proteins or nucleic acid macromolecules, leading to enzyme inactivation and toxic effects on cells [9], which further leads to damage to the cell membrane structure, decreases in selective permeability, inward flows of large amounts of substances, and increases in relative electrical conductivity. Moreover, under cold stress, plant cells are stimulated to produce many reactive oxygen species (ROS), mainly hydrogen peroxide (H_2_O_2_), hydroxide ions (OH^−^), hydroxyl radicals (-OH), and superoxide anions (O^2−^) [10, 11], which can damage cells. However, plants can protect themselves from damage through enzymatic and nonenzymatic antioxidant machinery systems [12]. The activity of antioxidant enzymes in tobacco plants first increases and then decreases under cold stress [13]. In addition to the physiological response, transcription factors are an important part of signalling and regulatory networks [14]. The transcription factor *ICE* (inducer of *CBF* expression) is rapidly expressed after low temperature induction and promotes the expression of *CBF* family genes, after which the *CBFs* bind to the CRT/DRE (C-repeat/dehydration response element) cis-element on the promoter of the *COR* (cold-regulated gene) to activate the expression of *COR* genes, which ultimately improves the cold tolerance of plants [8]. Studies have found that overexpression of *CBF1-3* enhances the expression of downstream *COR* genes and improves cold tolerance in *Arabidopsis* [15]. Another type of low-temperature signalling is the *CBF*-independent pathway. In *cbf* mutants of *Arabidopsis*, only 10%–20% of *COR* expression is significantly affected [16, 17]. Overexpression of the *MYB4* gene in rice can increase its cold resistance, but this process does not affect the expression of *CBF* [18]. Mutations in the *HOS9* gene in Arabidopsis can also cause plants to lose cold tolerance, which does not alter the expression of the *CBF* and *COR* genes [19].

BASIC PENTACYSTEINE (BPC) is a class of plant-specific transcription factors first identified in soybean and contains five conserved CYSTEINE residues in the C-terminal domain [20, 21]. It is also known as GAGA-binding protein (GBP) because of its ability to bind GAGA repeats specifically to regulate the expression of downstream genes [21, 22]. Currently, seven *BPC* genes have been identified in Arabidopsis and classified into three categories, including class I (*BPC1–BPC3*), class II (*BPC4–BPC6*), and class III (*BPC7*). Among them, *BPC5* is a pseudogene without coding function [21], and the other six *BPC* genes are all activators or repressors of their target genes [23]. However, in cucumber, four BPC members have been identified and have been divided into two categories: class I (*CsBPC1*-*CsBPC2*) and class II (*CsBPC3*-*CsBPC4*). *BPC* transcription factors play an important role in regulating plant growth and development [24]. It has been found that overexpression of *CsBPC2* in tobacco inhibits seed germination [20]. The *BPC* protein is also involved in the regulation of lateral root development, floral organ development, long-horn fruit elongation, and ovule development [23, 25]. In addition, *BPCs* are also involved in the regulation of hormones, such as cytokinin [26, 27], ethylene [23], and brassinosteroids [28]. *CsBPC2* is the most highly expressed *CsBPC* gene under various types of abiotic stresses and hormone treatments [20]. Recently, a study found that *BPC2* knockout decreased the salt tolerance of tobacco [20] and cucumber [24]. Therefore, BPC is involved not only in the regulation of plant growth and development but also in the regulation of abiotic stress.

Our previous study showed that the expression of Cs*BPC2* significantly increased during cold stress in cucumber [20]. However, whether *BPC2* is essential in cold tolerance and how *BPC2* regulates cold responses in plants are still unknown. In this study, by comparing WT and *Csbpc2* mutants under cold stress, we found for the first time that *CsBPC2* knockout significantly reduced the cold tolerance of cucumber. This study provides a reference for research on the biological functions of *BPC2* in other plants under cold stress and a theoretical basis for genetic engineering to breed low-temperature-tolerant cucumber varieties.

## Materials and methods

### Materials and treatments

Cucumber of WT (‘Changchunmici’) and two *Csbpc2* mutant lines (-54 bp, L2; -31 bp, L3) in which *Csbpc2* was knocked out by CRISPR/Cas9 [24] were used in this study. The seeds of the WT and two *Csbpc2* mutants of the same size and fullness were selected and immersed in water at 55 °C for 30 min and placed on a petri dish with two layers of filter paper in an incubator at 28 °C for germination. The germinated seeds were sown in a seedling pot (7 cm × 7 cm). The substrate (peat:vermiculite:chicken manure = 4:2:1, V:V:V) was added to fill the pot and watered thoroughly, and the plants were grown in a controlled greenhouse. Cucumber seedlings were grown under 14-h light (25 °C, 600 μmol m^−2^ s^−1^)/10-h dark (18 °C) photoperiods with 60%-70% air relative humidity. After approximately 25 days, the seedlings were treated with continuous cold at a temperature as low as 4 °C. This temperature and time were chosen because our previous preliminary experiments showed that the phenotype between WT and *Csbpc2* mutant cucumber seedlings was the most different. Furthermore, the activity of antioxidant enzymes had changed significantly after 6 h of cold stress. Therefore, samples were collected at 0, 6 and 24 h on the first leaf for the analysis of relative electrical conductivity, while the other indices were analyzed using samples obtained at 0 and 6 h. Samples were then put into an ultra-low-temperature freezer at -80 °C for subsequent experimental measurements.

### Methods

#### Measurement of the chilling injury index

The measurement of the chilling injury index was based on the method described in [29] with slight modifications. Each treatment had four biological replicates with four seedlings for each biological replicate. The leaves of cucumber seedlings were investigated according to the following classification criteria. Grade 0: cotyledons and true leaves were intact without obvious injury symptoms; Grade 1: one cotyledon was withered; Grade 2: both cotyledons were withered, and two true leaves appeared with few dehydration spots; Grade 3: two cotyledons and the heart leaf were withered, and the area of dehydration spots on the true leaves reached half of the whole leaf area; Grade 4: the area of dehydration spots on true leaves and heart leaves was more than half of the leaf area; and Grade 5: the whole plant was dehydrated and wilted.

$$\mathrm{Chilling}\;\mathrm{injury}\;\mathrm{index}=\frac{\sum\;\mathrm{Number}\;\mathrm{of}\;\mathrm{Plants}\;\mathrm{of}\;\mathrm{per}\;\mathrm{Grade}\;\times\;\mathrm{Corresponding}\;\mathrm{Grade}\;}{\mathrm{Number}\;\mathrm{of}\;\mathrm{Total}\;\mathrm{Plants}\;\times\;\mathrm{Highest}\;\mathrm{Grade}}\times100\%$$

#### Measurement of relative electrical conductivity

The relative electrical conductivity was measured according to the method described by Yan, 2019 [30]. We took the first leaf of cucumber and rinsed it once with tap water and then three times with distilled water. The water on the blades was blotted out as much as possible using clean filter paper. Next, 0.1 g was weighed, and the leaves were cut into 5 mm lengths with scissors. The cut leaves were then placed into a centrifuge tube and soaked in 10 ml of distilled water at 32 °C for 2 h. Following this, the initial conductivity (EC1) of the solution was measured using an electrical conductivity meter. Then, the immersion solution was placed in a boiling water bath for 20 min and cooled to 25 °C before measuring the final conductivity (EC2). The electrical conductivity of distilled water (EC3) was also determined. Relative electrical conductivity (EC) was calculated using the formula EC = (EC1-EC3)/(EC2-EC3) × 100%. There were three biological replicates for each treatment, and a random seedling was selected for each replicate.

#### Measurement of malondialdehyde (MDA) content

MDA levels were quantified via the thiobarbituric acid (TBA) reaction. The reagent (Suzhou Comin Biotechnology Co., Ltd, Suzhou, China) was added to 0.1 g of the prepared sample according to the manufacturer’s instructions, and the MDA content was then measured on a spectrophotometer. There were three biological replicates for each treatment, and two random seedlings were selected for each replicate.

#### Measurement of antioxidant enzyme activity

The activity of catalase (CAT) and peroxidase (POD) was measured by UV spectrophotometry, and the activity of superoxide dismutase (SOD) was measured by the nitroblue tetrazolium (NBT) method. The reagents were added according to the manufacturer’s instructions. All kits were provided by Suzhou Comin Biotechnology Co., Ltd, Suzhou, China. There were four biological replicates for each treatment, and a random seedling was selected for each replicate.

#### Transcriptome sequencing

Each treatment was set up with three biological replicates for transcriptome sequencing. Upon the isolation of total RNA from cucumber leaves, the purity and concentration of RNA were measured by a NanoDrop 2000 spectrophotometer. The RNA integrity was precisely assessed with Agient2100/LabChip GX. When the sample was qualified, library construction was initiated. After library construction was completed, a Qubit 3.0 fluorescence quantifier was used for preliminary quantification. The criterion was a concentration greater than 1 ng/µl. Next, a Qsep400 high-throughput analysis system was used to assess the inserted fragments of the library. When the inserted fragments met expectations, qPCR was used to accurately quantify the effective concentration of the library (effective concentration of the library > 2 nM) to ensure the quality of the library. Once the quality of the library had been confirmed, the Illumina NovaSeq 6000 sequencing platform was used for PE150 mode sequencing (Baimke Biotechnology Co., Ltd.). Then, the resulting raw data were filtered, and high-quality clean reads were obtained. These clean reads were then aligned to the cucumber reference genome by using TopHat software. The fragments per kilobase of transcript per million fragments mapped (FPKM) method was used to measure gene expression, and DESeq2 was used to screen differentially expressed genes. The filtering standard was set as log_2_|Fold Change|≥ 2 and FDR < 0.05. ClusterProfiler was used to perform Gene Ontology (GO) enrichment analyses of differentially expressed genes (https://github.com/GuangchuangYu/clusterProfiler/issues). Hypergeometric tests were used to identify GO terms that were significantly enriched for differentially expressed genes compared to the whole genomic background.

#### Measurement of gene expression

To further verify the accuracy of the sequencing data, we incorporated the techniques of quantitative real-time PCR (qRT‒PCR). We chosen six genes related to cold stress and randomly selected four differentially expressed genes. First, RNA isolation was carried out by using the Quick RNA Isolation Kit (Beijing Hua Yue Yang Biotechnology Co., Ltd.) from the prepared samples. The integrity of the RNA was evaluated by 1% agarose gel electrophoresis, and subsequently, the concentration of RNA was determined by using a BioLion spectrophotometer (BioLion Technology). A SuperScript Eraser cDNA Synthesis Kit (Beijing Hua Yue Yang Biotechnology Co., Ltd.) was employed for reverse transcription to obtain first-strand cDNA. After tenfold dilution of the first strand of the resulting cDNA, the diluted cDNA was used as a template for amplification. We used Primer Premier 5.0 software to design the primers. The primers were synthesized by Shanghai Biotech Co., Ltd., and real-time PCR mix (SYBR Green) (Beijing Ju He Mei Biotechnology Co., Ltd.) was used to perform real-time quantitative fluorescent qRT‒PCR amplification on an Mx3000P real-time quantitative fluorescent PCR instrument (Agilent Technologies). *CsActin* was used as the reference gene. The gene IDs used in the experiment and their primers are shown in Table 2. Based on the obtained Ct values, the relative expression of the target genes under different treatments was calculated by the 2^− ΔΔCt^ method. There were three biological replicates for each treatment, and a random seedling was selected for each replicate.

### Data analysis

The Tukey method (IBM SPSS 22.0 software) was utilized to determine the significance of differences in the data (α = 0.05, indicated in lowercase letters). GraphPad Prism 10.0 software was used to generate figures.

## Results

### Effects of *CsBPC2* knockout on the phenotype of cucumber seedlings under cold stress

**Fig. 1:**
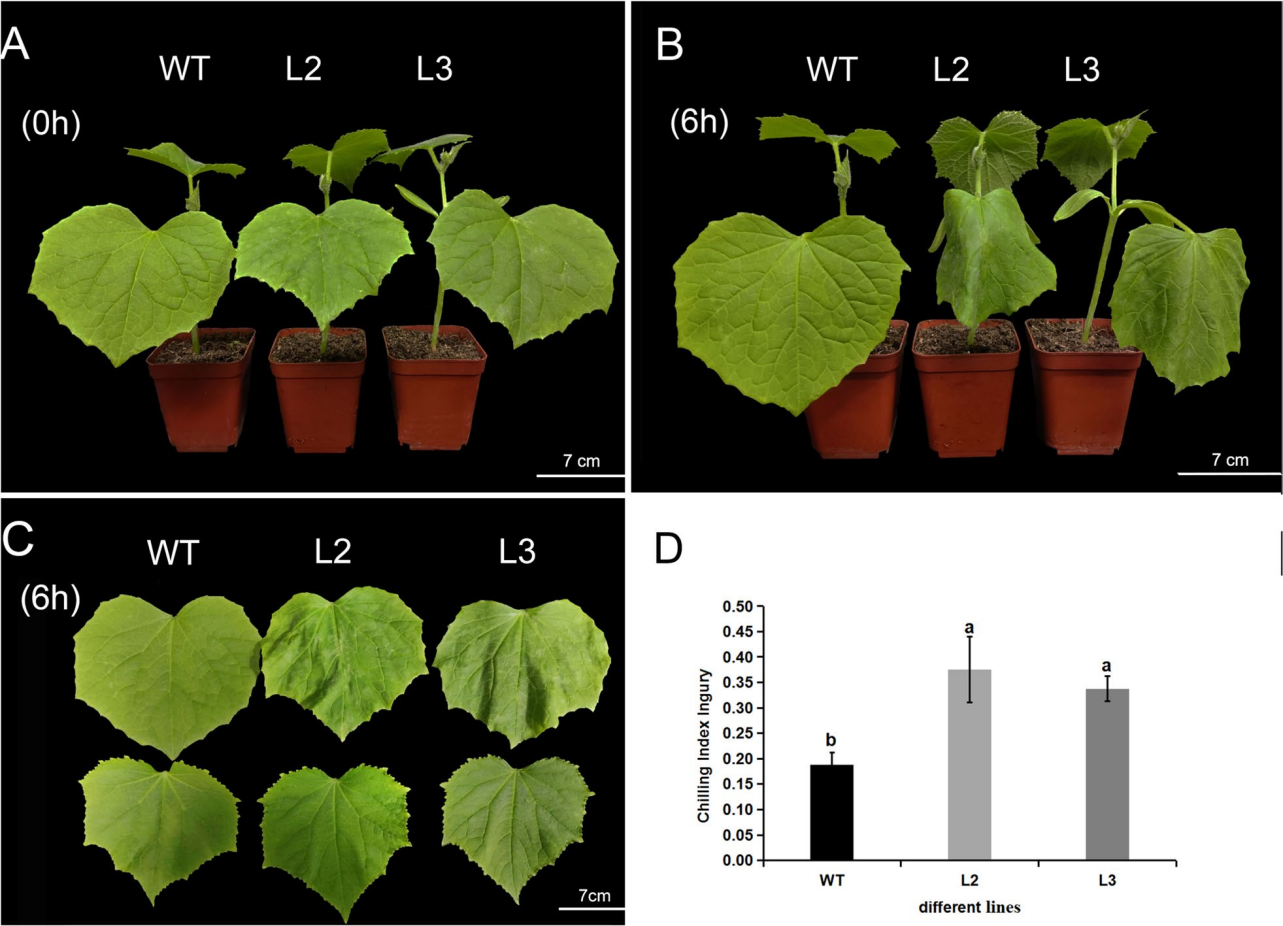
Effects of *CsBPC2* knockout on the phenotype and chilling injury index of cucumber seedlings under cold stress. **A** Phenotype of untreated cucumber plants. **B**-**C** Phenotype of cucumber plants treated at 4 °C for 6 h. **D** Chilling injury index of cucumber seedlings treated at 4 °C for 6 h. WT: wild-type cucumber seedlings; L2: cucumber *Csbpc2* mutant line L2; L3: cucumber *Csbpc2* mutant line L3. The different letters indicate significant differences as indicated by Tukey’s test (*p* < 0.05)

Before treatment (0 h) (Fig. 1A), the sizes of the WT and *Csbpc2* mutants were similar. However, after 6 h of cold stress at 4 °C, the leaves of *Csbpc2* mutants were more wilted and droopy than those of the WT (Fig. 1B-C). The chilling injury index values of the *Csbpc2* mutant lines L2 and L3 were 2- and 1.8-fold higher, respectively, than that of the WT (Fig. 1D). These results implied that the *Csbpc2* mutants were much more sensitive to cold stress than the WT control. Cold stress caused more serious damage to the *Csbpc2* mutant cucumber plants than to the WT plants.

### Effects of *CsBPC2* knockout on the relative electrical conductivity and MDA content of cucumber seedlings under cold stress

**Fig. 2:**
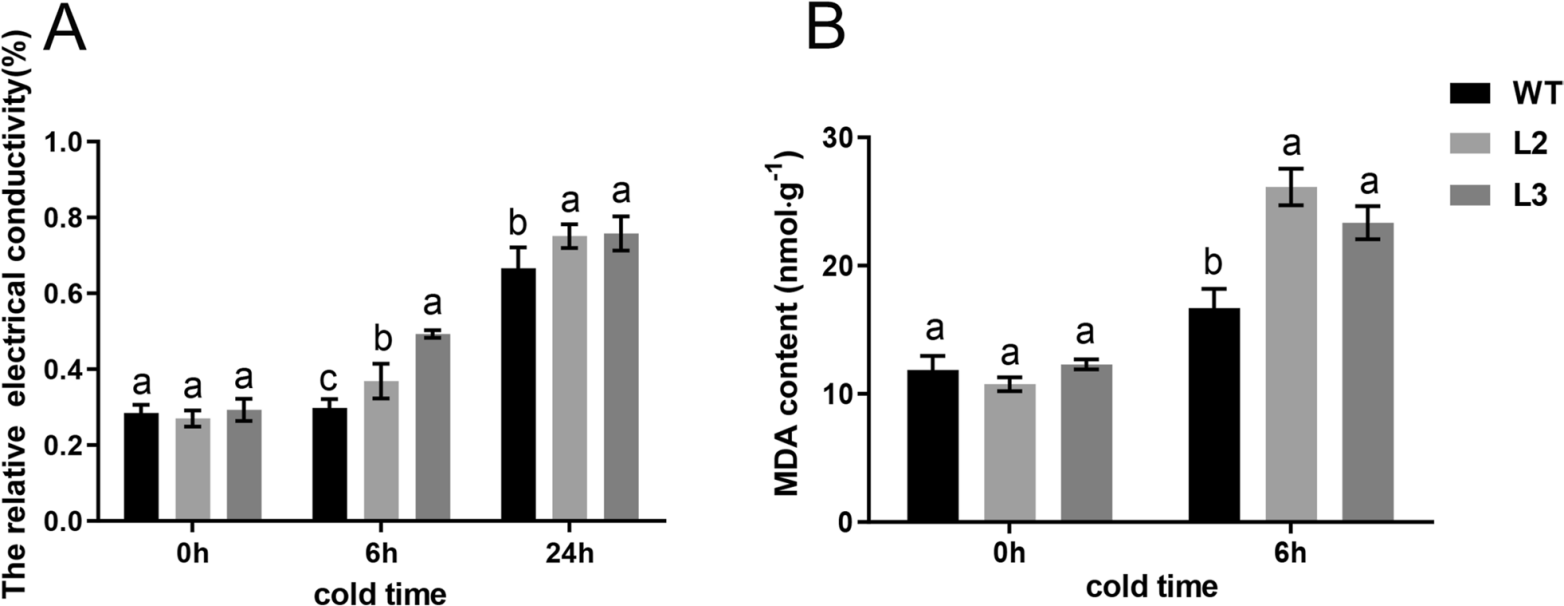
Effects of *CsBPC2* knockout on the relative electrical conductivity and MDA content of cucumber seedlings under cold stress. **A** Relative electrical conductivity. **B** MDA content. The different letters indicate significant differences tested by Tukey’s test (*p* < 0.05)

According to the data presented in Fig. 2, there was no significant difference in relative electrical conductivity or MDA content between WT and *Csbpc2* mutants under normal conditions. In contrast, when cucumber seedlings were exposed to cold stress at 4 °C, both the relative electrical conductivity and MDA content gradually increased in all plants, with significantly greater increases in the *Csbpc2* mutants L2 and L3 than in the WT control. After 6 h of cold stress, the relative electrical conductivity of the mutant lines L2 and L3 were 27.7% and 70.6% greater than that of the WT, respectively. After 24 h of cold stress, the relative electrical conductivity of the mutant plant lines L2 and L3 increased by 12.8% and 13.8%, respectively (Fig. 2A). Similarly, the MDA content of the mutant lines L2 and L3 was 58.5% and 39.9%, respectively, after 6 h of cold stress than in the WT control (Fig. 2B). Therefore, *CsBPC2* knockout led to weakened membrane stability of cucumber seedlings under cold stress.

### Effects of *CsBPC2* knockout on the activity of antioxidant enzymes in cucumber seedlings under cold stress

**Fig. 3:**
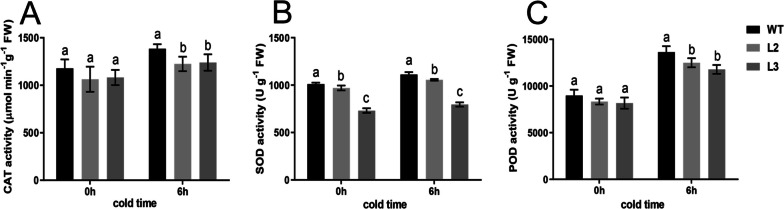
Effects of *CsBPC2* knockout on the activity of the antioxidant enzymes CAT (**A**), SOD (**B**) and POD (**C**) in cucumber seedlings under cold stress. The different letters indicate significant differences tested by Tukey’s test (*p* < 0.05)

Under normal growth conditions, there was no significant difference in the activity of the CAT and POD enzymes between the WT and *Csbpc2* mutants (Fig. 3A,C). After cold stress, the enzyme activity of CAT, SOD, and POD in the leaves of WT and mutant plants showed an upward trend. Nevertheless, the antioxidant enzyme activity in L2 and L3 was significantly lower than in the WT control (Fig. 3). After 6 h of cold stress treatment, the CAT activity of the WT was 13.1% and 12.0% than that of mutants L2 and L3, respectively (Fig. 3A). The SOD activity of the WT was 5.3% and 39.7% greater than that of mutants L2 and L3, respectively (Fig. 3B). The POD activity of WT was 9.1% and 16.0% greater than that of mutants L2 and L3, respectively (Fig. 3C). These results indicated that *CsBPC2* knockout significantly inhibited the antioxidant enzyme activity induced by cold stress, reduced the ability of plants to clear reactive oxygen species, and resulted in a weaker cold stress tolerance of seedlings.

### Transcriptome data quality analysis

The findings presented above showed that cucumber seedlings with *Csbpc2* mutations exhibited reduced tolerance to cold stress, both in terms of their phenotype and in terms of their physiological indicators. Then, we explored the changes at the transcriptome level. Transcriptome sequencing was performed on the leaves of the cucumber WT and the *Csbpc2* mutants L2 and L3, which were treated at 4 °C for 0 h and 6 h, respectively. From a total of 18 samples, we obtained 110.94 Gb of clean data. The clean data of each sample reached 5.74 Gb, and the percentage of Q30 bases was 93.12% or more. Clean reads of each sample were sequentially compared with the reference genome of *Cucumis sativus. ChineseLong_v2.genome.fa*, and the alignment efficiency was 92.25% to 96.88% (Table 1). In summary, the quality of the database was satisfactory.

**Table 1:** Statistical criteria of the transcriptome sequencing data

Samples	Total reads	Clean reads	Clean bases	GC content	% ≥ Q30	Mapping ratio
0 h-WT-1	46,698,690	23,349,345	6,987,035,978	44.34%	94.65%	96.70%
0 h-WT-2	39,832,516	19,916,258	5,961,421,700	44.23%	94.32%	96.82%
0 h-WT-3	40,442,870	20,221,435	6,052,180,658	44.53%	94.68%	96.16%
0 h-L2-1	39,648,528	19,824,264	5,931,364,386	44.46%	94.83%	96.52%
0 h-L2-2	42,377,044	21,188,522	6,342,339,252	44.23%	94.67%	96.88%
0 h-L2-3	40,898,050	20,449,025	6,118,997,538	44.80%	93.83%	96.19%
0 h-L3-1	40,363,938	20,181,969	6,039,962,410	43.98%	93.12%	96.21%
0 h-L3-2	42,798,128	21,399,064	6,403,580,358	45.02%	94.70%	92.25%
0 h-L3-3	41,483,912	20,741,956	6,205,228,358	44.10%	94.39%	96.67%
6 h-WT-1	38,373,648	19,186,824	5,740,142,498	44.02%	94.46%	96.48%
6 h-WT-2	40,552,436	20,276,218	6,066,290,918	44.15%	94.49%	96.64%
6 h-WT-3	40,282,554	20,141,277	6,027,263,606	43.96%	94.10%	96.58%
6 h-L2-1	40,669,314	20,334,657	6,083,389,134	44.12%	93.87%	96.42%
6 h-L2-2	40,166,458	20,083,229	6,009,679,630	44.09%	93.39%	96.22%
6 h-L2-3	41,971,962	20,985,981	6,277,400,236	44.26%	94.80%	96.65%
6 h-L3-1	39,805,680	19,902,840	5,955,646,082	44.18%	94.26%	96.53%
6 h-L3-2	45,255,956	22,627,978	6,765,563,802	43.81%	94.47%	96.74%
6 h-L3-3	39,940,984	19,970,492	5,976,063,820	44.01%	94.05%	96.85%

### Differential gene expression patterns under cold stress

**Fig. 4:**
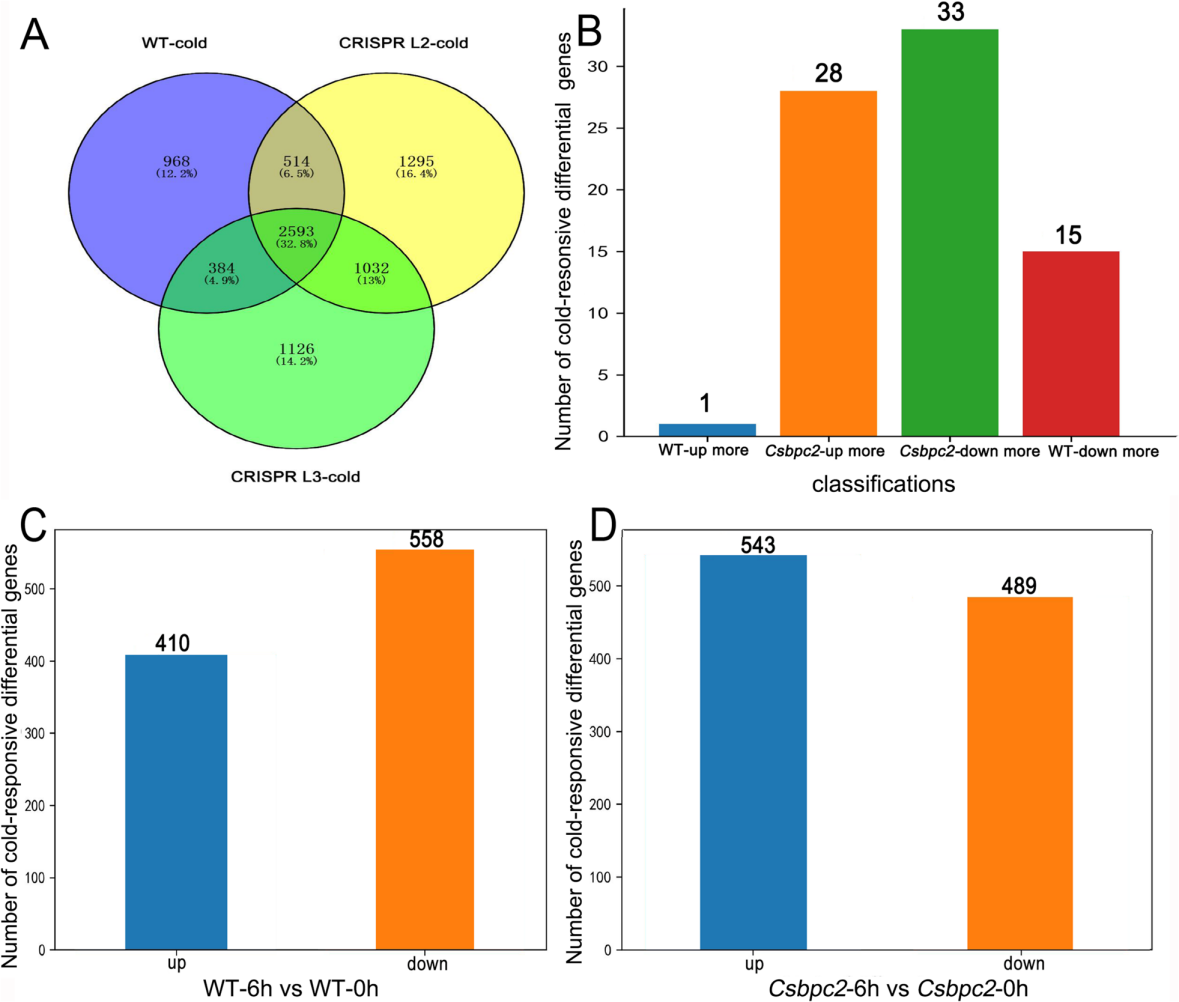
Number of differential genes responsible to cold stress in the WT and *Csbpc2* mutants. **A** Venn diagram of cold-responsive differentially expressed genes. **B** Classification of common cold-responsive differentially expressed genes in the WT and *Csbpc2* mutants (WT-up more: the expression of the genes increased more in the WT than in mutants. *Csbpc2*-up more: the expression of the genes increased more in the mutants than in the WT. *Csbpc2*-down more: the expression of the genes decreased more in the mutants than in the WT. WT-down more: the expression of the genes decreased more in the WT than in mutants) (log2FC ≥ 2). **C** Number of unique up-/down-regulated cold-responsive differentially expressed genes in the WT. **D** Number of unique up-/down-regulated cold-responsive differentially expressed genes in the *Csbpc2* mutants

To understand how gene expression patterns changed in the WT and *Csbpc2* mutants under cold stress, we analysed transcriptional data. As shown in Fig. 4A and Table S1, there were 2593 common differentially expressed genes shared by the WT and *Csbpc2* mutants (L2, L3) under cold stress. Only 77 genes showed significant differences between the WT and *Csbpc2* mutants, which could be divided into four categories (Fig. 4B; Fig. S1). One large category contained the genes whose expression was up-regulated after cold stress, but the expression in *Csbpc2* mutants increased more than that in the WT (28 genes). The other large category contained genes whose expression was down-regulated after cold stress, but the expression in *Csbpc2* mutants decreased more than that in the WT (33 genes). Moreover, there were only 968 cold-responsive differentially expressed genes unique to the WT, including 410 up-regulated genes and 558 down-regulated genes (Fig. 4C; Fig. S2, Table S2). The *Csbpc2* mutant L2 line had 1295 unique cold-responsive genes, while the *Csbpc2* mutant L3 line had 1126. There were 1032 common cold-responsive genes between the two mutant lines, of which 543 were up-regulated and 489 were down-regulated (Fig. 4D; Fig. S3; Table S3). Interestingly, the number of unique cold-responsive genes in *Csbpc2* mutants was higher than that in the WT. All these results indicated that *CsBPC2* knockout resulted in more cold-responsive genes under cold stress than were in the WT.

### GO enrichment analysis of differentially expressed genes

**Fig. 5:**
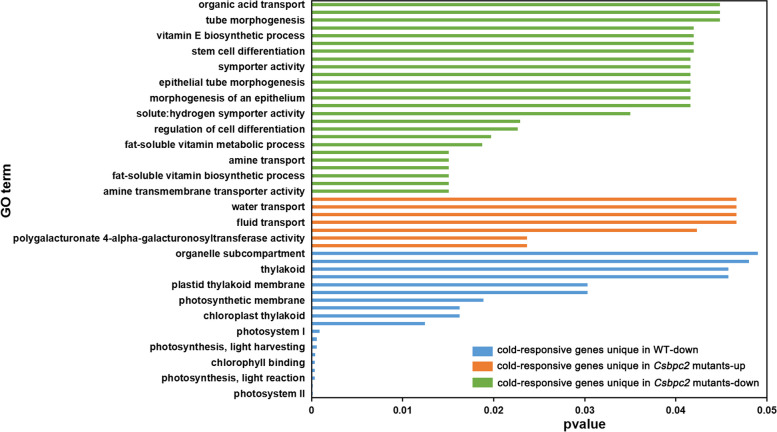
GO analysis of cold-responsive differentially expressed genes unique to the WT (blue) or *Csbpc2* mutants (orange and green)

**Fig. 6:**
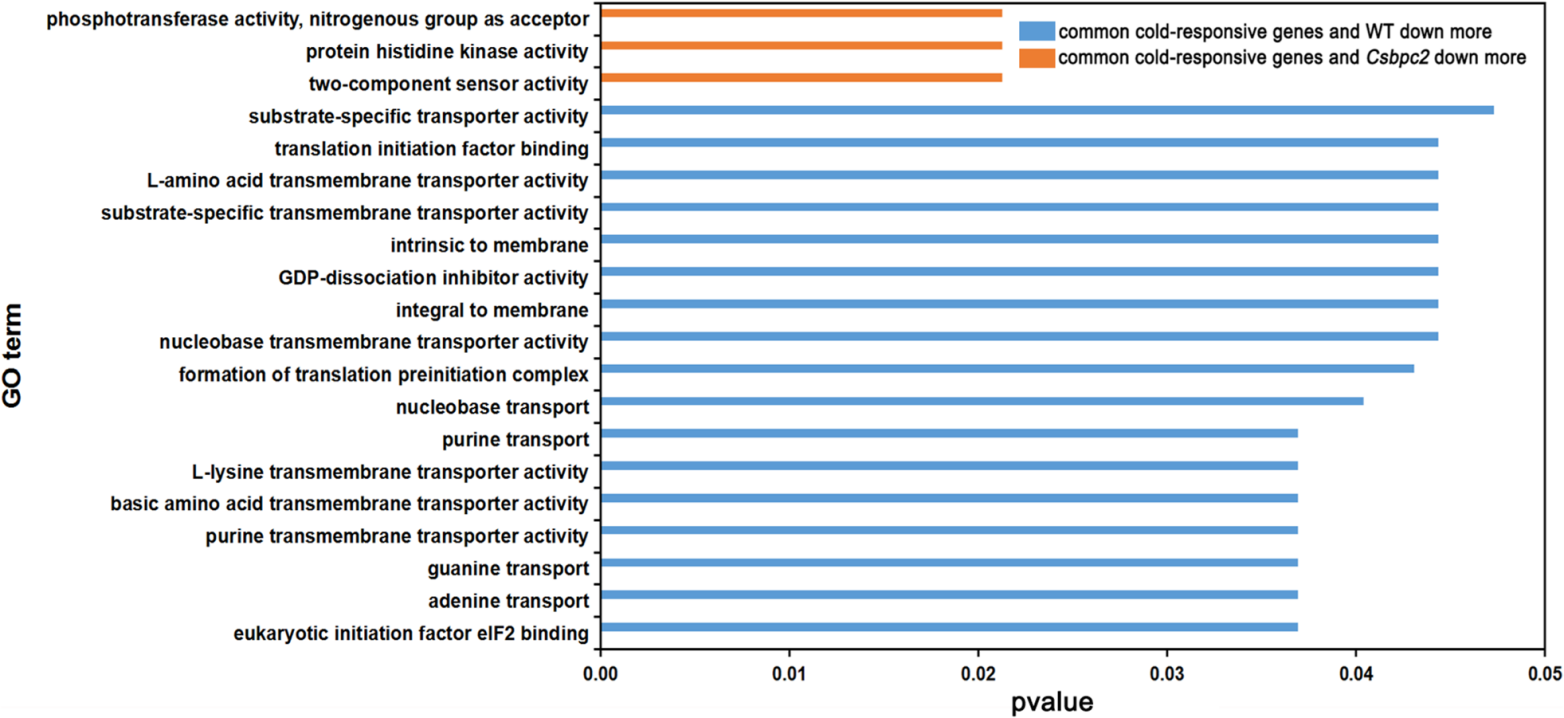
GO analysis of common cold-responsive differentially expressed genes in both the WT (blue) and *Csbpc2* mutants (orange)

To understand the potential functions of the cold-responsive differentially expressed genes, GO enrichment analysis was carried out. As shown in Fig. 5 and Table S4, GO analysis showed that the up-regulated genes unique to the mutants were related to biosynthesis. Nevertheless, the main enriched GO term was associated with down-regulated cold-responsive genes, whose functions were mainly related to amino acid transport, vitamin metabolism and transport, etc. These GO results suggest that *CsBPC2* knockout affects plant tolerance to cold stress in different ways. However, it seems that the down-regulation of the genes related to the biosynthesis of amino acids and vitamins could negatively affect cold tolerance in cucumber plants. The down-regulated genes unique to the WT were related mainly to photosynthesis. This indicated that cold stress could affect plant photosynthesis. In addition to analysing the function of genes specific to the WT and specific to *Csbpc2* mutations, we also analysed common differentially expressed genes between the WT and *Csbpc2* mutants (Fig. 6). The common cold-responsive differentially expressed genes between the WT and *Csbpc2* mutants were enriched mainly in two major categories. In one category, these genes were down-regulated, and the expression in the WT was down-regulated more than that in *Csbpc2* mutants. The functions of these genes were related mainly to purine transport and transporter protein activity. In the other category, the genes were down-regulated, and the expression in *Csbpc2* mutants was down-regulated more than that in the WT. The functions of these genes were related mainly to the activity of protein histidine kinase, phosphotransferase, etc. The results showed that in *Csbpc2* mutants and the WT, plants could also reduce the expression of purine transport, transporter, protein histidine kinase and phosphotransferase genes to affect their cold tolerance.

### *CsBPC2* was essential for the expression of some key cold-responsive genes

**Fig. 7:**
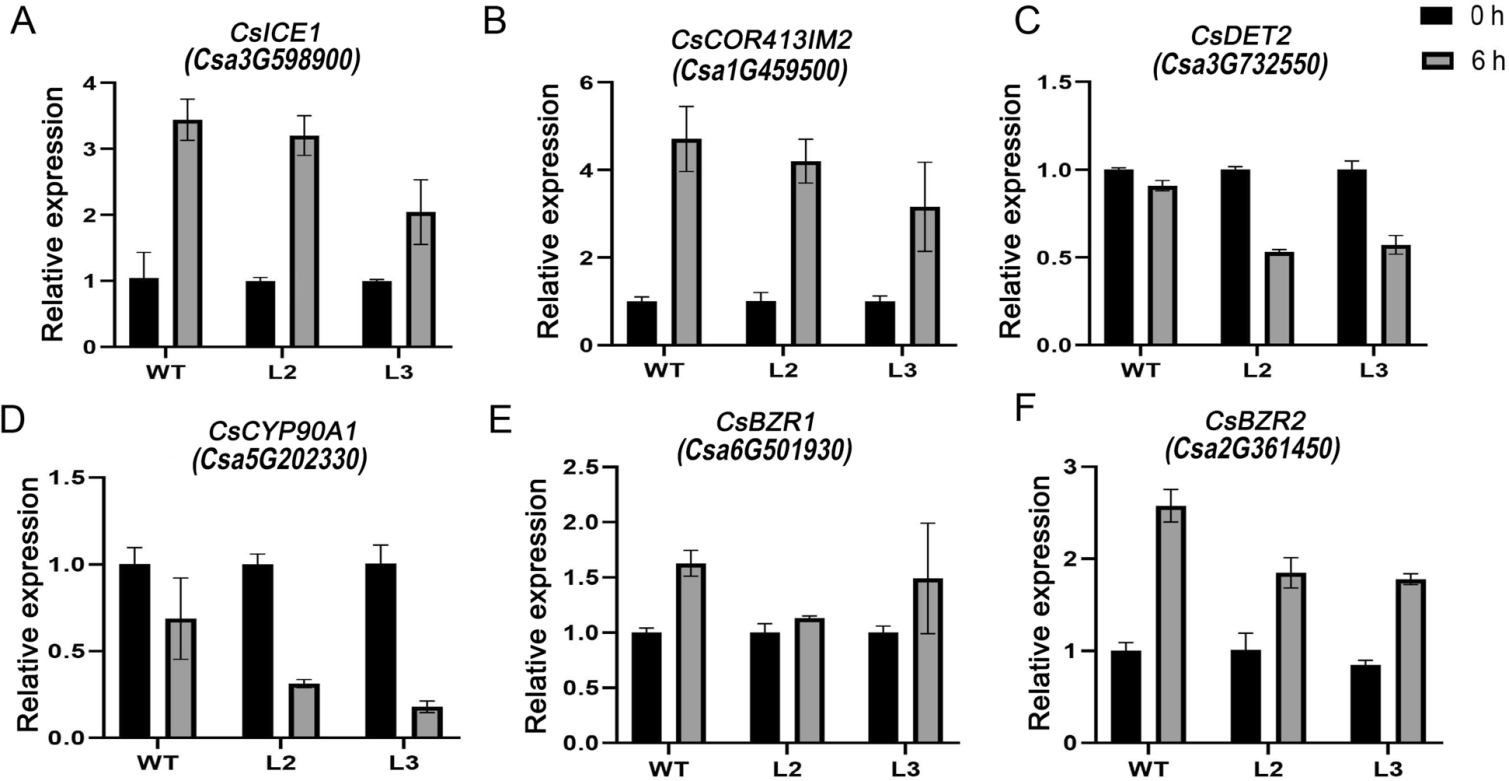
Relative expression (2^-ΔΔCt^) of the cold-responsive genes

To verify the accuracy of the transcriptome sequencing results, we randomly selected four differentially expressed genes and six genes that played an important role in the cold response process for qRT‒PCR verification (Table 2). Compared with the RNA-seq results, we found that the differential gene expression results tended to coincide, indicating that the sequencing results had high accuracy and reliability (Fig. 7; Fig. S4). Among the ten differentially expressed genes, the six cold-responsive genes involved the *CBF*-dependent pathway and BR signal synthesis and transduction genes, which are key genes regulating plant cold tolerance. The relative expression of *CsICE1* (*Csa3G598900*), *CsCOR413IM2* (*Csa1G459500*), *CsBZR1* (*Csa6G501930*) and *CsBZR2* (*Csa2G361450*) was up-regulated after cold stress. The relative expression of *CsDET2* (*Csa3G732550*) and *CsCYP90A1* (*Csa5G202330*) was down-regulated after cold stress. However, compared with the WT plants, *CsBPC2*-knockout plants exhibited significantly reduced expression of these genes, indicating the importance of *CsBPC2* for cold tolerance in cucumber.

**Table 2:** Differentially expressed genes in the WT and *Csbpc2* mutants used for analysis of qRT‒PCR

Gene ID	WT-log2 fold Change	L2-log2 fold Change	L3-log2 fold Change	WT-regulation	L2-regulation	L3-regulation	Forward primer Sequence (5'-3')	Reverse primer Sequence (5'-3')
*Csa3G598900*	1.160573749	0.71314422	0.970121949	up	up	up	TTTAGGCGGTGAAAATGGTC	CACAGGCATCGAAGTTCTCA
*Csa1G459500*	0.485592215	0.148236934	0.037536613	up	up	up	GTTGTGCTCCGTCTCTTCTT	TCCGATGGGATCGTTTCTTATG
*Csa3G732550*	-0.760215992	-0.103116865	-0.022952602	down	down	down	AGAAGTCATTTGTGACAATGGC	CAATACCAATAGCTCCGACTCT
*Csa5G202330*	-1.089579003	-1.509829003	-1.514338257	down	down	down	TCTTCATCATCTTGTCACCCAA	TCGTTCTTACGCGTCACATATA
*Csa6G501930*	1.271882853	1.057699327	1.258896149	up	up	up	CGAAGCAAAGAAGCAAAACATC	TCAAGCTAAAATGTTCGGTTCG
*Csa2G361450*	0.364259096	0.216829221	0.243954024	up	up	up	CTCTCTCTCTTTCCCAATCGAA	CGATTCTTCAGTTCCGACAATC
*Csa3G643770*	0.777739139	1.741103697	1.712188562	up	up	up	GGAGAGTTCAGCCGTGACAGTAGA	ACACATCCTTCTTCCTGACCCAAATG
*Csa7G407550*	0.803906259	1.796374033	1.798755046	up	up	up	CGATAACTCCGTCTCCGTCCTCTT	GAACAACTCTCCGATCAACCTCACTC
*Csa1G533380*	-0.631431667	-1.412083745	-1.363809409	down	down	down	TTGGTGTGATTCAAGGCTCTAT	TCCGTCGCCATATAAGCATAAT
*Csa2G369740*	-0.765350909	-1.584917728	-1.810774018	down	down	down	AACAAAAGTTCACGAGCTACAC	CTGCTATGTACTCTCCATTCGT
*CsActin*	/	/	/	/	/	/	TTCTGGTGATGGTGTGAGTC	GGCAGTGGTGGTGAACATG

## Discussion

### *CsBPC2* may positively regulate the cold tolerance of cucumber

Cold has been an important factor limiting the yield of cucumber in protected cultivation [1]*.* The signal sensing and response model of plants to cold stress was established on the basis of extensive research on Arabidopsis [31]. However, research on the molecular mechanisms of the *Cucurbitaceae* family in response to cold stress is weak compared with that on model plants. Further detailed studies are needed to understand the mechanisms by which signals are received and transmitted, as well as plants’ response to cold [32, 33].

*BPCs* are homologous to the GBP (GAGA binding protein) protein in soybean [22]. Several studies have shown that *BPCs* are of vital importance in the regulation of plant growth and development. They participate in processes within the internodes, leaves, flowers [23, 26, 34], ovules, embryos [21, 35, 36], and seeds [23] of *Arabidopsis* as well as in other regulatory processes. Our previous research found four *BPC* genes in cucumber that were classified into two groups. These four types of *CsBPCs* were all widely expressed in both nutritional and reproductive organs. In addition, different abiotic stresses and hormonal treatments could induce the expression of *CsBPCs* [20]. Overexpression of *CsBPC2* in tobacco inhibited the germination of seeds under saline, polyethylene glycol, and abscisic acid (ABA) conditions [20]. *CsBPC2* is also essential in the salt tolerance of cucumber [24]. These results suggest that *CsBPCs* may actively participate in regulating abiotic stress and phytohormones in cucumber. The function of *BPCs* in plant resistance to cold stress has not been reported. In this study, *Csbpc2* mutants were used to investigate the function of *CsBPC2* in cold tolerance. We found that the loss of *CsBPC2* increased the sensitivity of cucumber seedlings to cold stress. The mutants suffered more severe water loss, greater wilting, and a higher chilling injury index than the WT (Fig. 1). MDA is an end product of membrane lipid peroxidation, which can damage the structure and function of the biological membrane and change the membrane permeability such that its content can reflect the degree of plant damage. Studies have shown that the amount of MDA increases in cucumber seedlings under cold stress [13]. Similarly, the MDA content and the relative electrical conductivity of *Csbpc2* mutants were significantly higher than those of the WT after cold stress in this study (Fig. 2), indicating that their membrane structure and cells were more seriously damaged and that their cell membrane permeability had increased, further leading to an outflow of intracellular electrolytes and an increase in relative electrical conductivity. The above results showed that knockout of *CsBPC2* reduced the cold tolerance of cucumber seedlings, which indicated that *CsBPC2* was essential for cold tolerance.

### *CsBPC2* participated in the regulation of the antioxidant pathway in cucumber seedlings under cold stress

Stress causes the accumulation of toxic substances and induces other stresses [37, 38]. Under normal conditions, the production and elimination of free radicals within cells are balanced. However, when subjected to cold stress, plants are stimulated to produce excessive amounts of reactive oxygen species (ROS). The balance of energy supply within the cell is disrupted. The massive accumulation of ROS, which possess strong oxidative capacity, leads to cellular damage [3, 12]. The activity of protective enzymes such as CAT, SOD, and POD in the plant will be increased to alleviate damage to plants under cold stress [39]. In this study, the enzyme activity of CAT, SOD, and POD in both the *Csbpc2* mutants and WT cucumber seedling leaves showed an increasing trend under cold stress. However, in the *Csbpc2* mutants, the enzyme activity was consistently lower than that in the WT, and the increase rate was also lower than that in the WT (Fig. 3), suggesting that *CsBPC2* was involved in the regulation process of the antioxidant enzyme pathway under cold stress.

### *CsBPC2* knockout decreased gene expression related to the synthesis and transport of amino acids and vitamins under cold stress

Plant adaptation to cold stress is a complex process that involves a series of changes at both physiological and molecular levels [32]. Transcriptome sequencing is a powerful technology capable of revealing the transcriptional activities of any species at the mononucleotide level [40, 41]. The results of the present study indicated that *CsBPC2* was involved in cold tolerance in cucumber. However, how *CsBPC2* knockout affected the global transcriptome under cold stress was not clear. In our study, the composition and expression of cold-responsive genes in cucumber seedlings significantly changed in *Csbpc2* mutants. Cold stress caused more gene changes in the *Csbpc2* mutants than in the WT (Fig. 4). Amino acids are osmoregulatory factors that maintain and stabilize cell structure and usually accumulate in plants in response to stress [42]. Our results showed that the down-regulated cold-responsive differentially expressed genes unique to *Csbpc2* mutants were significantly enriched. The functions of these genes were related mainly to amino acid transport, vitamin metabolism, and transport (Fig. 5). Thus, the functional loss of *CsBPC2* led to decreases in amino acid transport, vitamin metabolism, and transport processes during cold stress, which may ultimately affect metabolism to reduce the cold tolerance of cucumber seedlings. In addition, interestingly, we found that the photosynthesis-related genes of WT decreased significantly under cold stress, while the photosynthesis-related genes of *Csbpc2* mutants showed no difference under cold stress (Fig. 5). While, our previous study showed that under normal conditions, knocking out *CsBPC2* improved the expression of photosynthesis-related genes [43], suggesting that *CsBPC2* negatively regulated the expression of these genes, which may be the main reason why there was no change in photosynthesis of *Csbpc2* mutants during cold stress compared to the WT in the present study. Another reason may be the stress applied in our experiment was too short to generalize the link between the expression of photosynthesis-related genes and *Csbpc2* mutants. In general, we are the first time found that knocking out *CsBPC2* mainly decreased the expression of molecules involving in amino acid transport, vitamin metabolism and *ICE-CBF* pathways that reduced the cold tolerance of cucumber seedlings.

### *CsBPC2* was involved in the regulation of important cold-responsive genes in cucumber seedlings under cold stress

At present, the expression of *CBF* pathway genes in plant hypothermia molecular regulatory mechanisms is of great significance for enhancing plant tolerance to cold stress [44, 45]. Upstream *CBF* genes are regulated by the transcription factor *ICE,* while downstream binding of the *COR* gene promoter activates *COR* gene expression, thereby improving plant cold tolerance [46]. Transgenic tomato plants possessing the *LeCBF1* gene exhibit greater cold tolerance than WT plants [47]. The expression of the *COR* gene is crucial for cold tolerance and low-temperature acclimation [48]. Overexpression of the *CBF* gene can increase *COR* gene expression and enhance plant cold tolerance [15, 49–51]. The expression of *COR413IM2* has been reported to be induced by cold [52]. In *Arabidopsis thaliana*, the expression of *COR413IM2* is significantly increased under cold stress [53]. We found that the expression of *CsICE1* and *CsCOR413IM2* was up-regulated after cold stress but that the expression in *Csbpc2* mutants was lower than that in the WT (Fig. 7A-B). In addition to the *CBF* regulatory pathway, brassinosteroids (BRs) also play an important role in regulating plant resistance to cold stress [54]. *DET2, CYP90A1, CsBZR1* and *BZR2* are all key regulators of the BR pathway, and a study has found that *BZR1* can positively regulate cold tolerance in plants [55, 56]. Our results showed that the expression of BR synthesis genes (*CsDET2, CsCYP90A1*) was down-regulated under cold stress and decreased more in *Csbpc2* mutants than in the WT (Fig. 7C-D). The expression of *CsBZR1* and *CsBZR2* increased, but the increase in mutants was less than that in the WT (Fig. 6E-F). In short, both RNA-seq and qPCR techniques indicated that *CsBPC2* regulated the expression of *CsICE1, CsCOR413IM2, CsDET2, CsCYP90A1, CsBZR1 and CsBZR2* and that *CsBPC2* knockout suppressed the expression of these genes to reduce cold tolerance in cucumber seedlings.

## Conclusion

*Csbpc2* mutant cucumber seedlings showed significant increases in chilling injury index, relative electrical conductivity, and MDA content after cold stress. The activity of the antioxidant enzymes CAT, SOD, and POD was significantly reduced. Moreover, *CsBPC2* knockout not only affected the expression of cold function genes but also decreased the expression of some key metabolic genes under cold stress. In conclusion, this study reveals for the first time that *CsBPC2* is essential for cold tolerance under cold stress.

### Supplementary Information

**Additional file 1.** **Table S1.** The common cold-responsive differential genes shared by WT and *Csbpc2* mutants

** Additional file 2.** **Table S2.** Cold-responsive differential genes unique to WT

** Additional file 3.** **Table S3.** Cold-responsive differential genes unique to *Csbpc2* mutants (L2, L3)

** Additional file 4.** **Table S4.** Transcriptome cold-responsive differential genes GO enrichment analysis

** Additional file 5.** **Figure S1.** Heat map of common cold-responsive differential genes between WT and *Csbpc2* mutants. A. Category one: the expression of the genes increased more in the WT than in mutants. B. Category two: the expression of the genes increased more in the mutants than in the WT. C. Category three: the expression of the genes decreased more in the mutants than in the WT. D. Category four: the expression of the genes decreased more in the WT than in mutants. **Figure S2.** Heat map of cold-responsive differential genes unique to WT. A. up-regulated genes; B. down-regulated genes. **Figure S3.** Heat map of cold-responsive differential genes unique to *Csbpc2* mutants (L2,L3). A. up-regulated genes; B. down-regulated genes. **Figure S4.** Relative expression (2^-ΔΔCt^) of the differentially expressed genes.

## Data Availability

The raw RNA sequence data have been submitted to the National Center for Biotechnology Information (NCBI) under accession number PRJNA1006375 (https://www.ncbi.nlm.nih.gov/sra/PRJNA1006375). All other data generated or analyzed during this study are included in this manuscript.

## Abbreviation

WT: Wild-type
MDA: Malondialdehyde
RNA-seq: RNA Sequencing
GO: Gene Ontology
qPT-PCR: Quantitative real-time polymerase chain reaction
*ICE*: Inducer of *CBF* expression
*CBF*: C-repeat/dehydration response element
*COR*: Cold-regulated gene
*BPC*: Basic pentacysteine
CAT: Catalase
SOD: Superoxide dismutase
POD: Peroxidase
L2: Mutant line L2
L3: Mutant line L3

## References

[1] Olechowska E, Słomnicka R, Kaźmińska K, Olczak-Woltman H, Bartoszewski G (2022). The genetic basis of cold tolerance in cucumber (Cucumis sativus L.)-the latest developments and perspectives. J Appl Genet.

[2] Huang JN, Zhao JY, Wang X, Ma LF, Ma ZT, Meng XN, Fan HY (2023). SnRK1 signaling regulates cucumber growth and resistance to *Corynespora cassiicola*. Plant Sci.

[3] Li CX, Dong SY, Bo KL, Miao H, Zhang SP, Gu XF. Research progress in physiological and molecular mechanism of low temperature stress response in cucumber. China Vegetables. 2019;(05):17–24(In Chinese).

[4] Guo XY, Liu DF, Chong K (2018). Cold signaling in plants: Insights into mechanisms and regulation. J Integr Plant Biol.

[5] Liu ZY, Jia YX, Ding YL, Shi YT, Li Z, Guo Y, Gong ZZ, Yang SH (2017). Plasma membrane CRPK1-Mediated phosphorylation of 14-3-3 proteins induces their nuclear import to Fine-Tune CBF signaling during cold response. Mol Cell.

[6] Kidokoro S, Shinozaki K, Yamaguchi-Shinozaki K (2022). Transcriptional regulatory network of plant cold-stress responses. Trends Plant Sci.

[7] Foyer CH, Noctor G (2005). Redox homeostasis and antioxidant signaling: a metabolic interface between stress perception and physiological responses. Plant Cell.

[8] Li JL, Li HM, Quan XY, Shan QL, Wang WB, Yin N, Wang SQ, Wang ZH, He WX (2022). Comprehensive analysis of cucumber C-repeat/dehydration-responsive element binding factor family genes and their potential roles in cold tolerance of cucumber. BMC Plant Biol.

[9] Rihan HZ, Al-Issawi M, Fuller MP (2017). Advances in physiological and molecular aspects of plant cold tolerance. J Plant Interact.

[10] Li SH, Liu S, Zhang Q, Cui MX, Zhao M, Li NY, Wang SN, Wu RG, Zhang L, Cao YP, Wang LH (2022). The interaction of ABA and ROS in plant growth and stress resistances. Front Plant Sci.

[11] Nadarajah KK (2020). ROS Homeostasis in abiotic stress tolerance in plants. Int J Mol Sci.

[12] Duan XY, Yu XJ, Wang YD, Fu W, Cao RF, Yang L, Ye XL (2022). Genome-wide identification and expression analysis of glutathione S-transferase gene family to reveal their role in cold stress response in cucumber. Front Genet.

[13] Anwar A, Liu YM, Dong RR, Bai LQ, Yu XC, Li YS (2018). The physiological and molecular mechanism of brassinosteroid in response to stress: a review. Biol Res.

[14] Chen LG, Song Y, Li SJ, Zhang LP, Zou CS, Yu D (2012). The role of WRKY transcription factors in plant abiotic stresses. Biochim Biophys Acta.

[15] Gilmour SJ, Fowler SG, Thomashow MF (2004). Arabidopsis transcriptional activators *CBF1*, *CBF2*, and *CBF3* have matching functional activities. Plant Mol Biol.

[16] Zhao CZ, Zhang ZJ, Xie SJ, Si T, Li YY, Zhu J-K (2016). Mutational evidence for the critical role of CBF transcription factors in cold acclimation in Arabidopsis^1^. Plant Physiol.

[17] Jia YX, Ding YL, Shi YT, Zhang XY, Gong ZZ, Yang SH (2016). The *cbfs* triple mutants reveal the essential functions of *CBFs* in cold acclimation and allow the definition of CBF regulons in *Arabidopsis*. New Phytol.

[18] Vannini C, Locatelli F, Bracale M, Magnani E, Marsoni M, Osnato M, Mattana M, Baldoni E, Coraggio I (2004). Overexpression of the rice *Osmyb4* gene increases chilling and freezing tolerance of *Arabidopsis thaliana* plants. Plant J.

[19] Zhu JH, Shi HZ, Lee B, Damsz B, Cheng S, Stirm V, Zhu JK, Hasegawa PM, Bressan RA (2004). An *Arabidopsis* homeodomain transcription factor gene, *HOS9*, mediates cold tolerance through a CBF-independent pathway. Proc Natl Acad Sci USA.

[20] Li SZ, Miao L, Huang B, Gao LH, He CX, Yan Y, Wang J, Yu XC, Li YS (2019). Genome-wide identification and characterization of cucumber BPC transcription factors and their responses to abiotic stresses and exogenous phytohormones. Int J Mol Sci.

[21] Meister RJ, Williams LA, Monfared MM, Gallagher TL, Kraft EA, Nelson CG, Gasser CS (2004). Definition and interactions of a positive regulatory element of the *Arabidopsis INNER NO OUTER* promoter. Plant J.

[22] Sangwan I, O'Brian MR (2002). Identification of a soybean protein that interacts with GAGA element dinucleotide repeat DNA. Plant Physiol.

[23] Monfared MM, Simon MK, Meister RJ, Roig-Villanova I, Kooiker M, Colombo L, Fletcher JC, Gasser CS (2011). Overlapping and antagonistic activities of *BASIC PENTACYSTEINE* genes affect a range of developmental processes in Arabidopsis. Plant J.

[24] Li SZ, Sun MT, Miao L, Di QH, Lv LJ, Yu XC, Yan Y, He CX, Wang J, Shi AK, Li YS (2023). Multifaceted regulatory functions of CsBPC2 in cucumber under salt stress conditions. Hortic Res.

[25] Mu Y, Liu YM, Bai LQ, Li SZ, He CX, Yan Y, Yu XC, Li YS (2017). Cucumber CsBPCs regulate the expression of CsABI3 during seed germination. Front Plant Sci.

[26] Simonini S, Kater MM (2014). Class I BASIC PENTACYSTEINE factors regulate *HOMEOBOX* genes involved in meristem size maintenance. J Exp Bot.

[27] Shanks CM, Hecker A, Cheng CY, Brand L, Collani S, Schmid M, Schaller GE, Wanke D, Harter K, Kieber JJ (2018). Role of *BASIC PENTACYSTEINE* transcription factors in a subset of cytokinin signaling responses. Plant J.

[28] Theune ML, Bloss U, Brand LH, Ladwig F, Wanke D (2019). Phylogenetic analyses and GAGA-motif binding studies of BBR/BPC proteins lend to clues in GAGA-motif recognition and a regulatory role in brassinosteroid signaling. Front Plant Sci.

[29] Di QH, Li YS, Li SZ, Shi AK, Zhou MD, Ren HZ, Yan Y, He CX, Wang J, Sun MT, Yu XC (2022). Photosynthesis mediated by *RBOH*-dependent signaling is essential for cold stress memory. Antioxidants (Basel).

[30] Yan Y. Functional analysis of low-temperature tolerance conferred by the GPA1-encoded G protein subunit in *Cucumis sativus*. China Agricultural University, thesis, China, 2019 (In Chinese).

[31] Zhu J-K (2016). Abiotic stress signaling and responses in plants. Cell.

[32] Ding YL, Shi YT, Yang SH (2019). Advances and challenges in uncovering cold tolerance regulatory mechanisms in plants. New phytol.

[33] Kopeć P, Rapacz M, Arora R (2022). Post-translational activation of CBF for inducing freezing tolerance. Trends Plant Sci.

[34] Santi L, Wang YM, Stile MR, Berendzen K, Wanke D, Roig C, Pozzi C, Müller K, Müller J, Rohde W, Salamini F (2003). The GA octodinucleotide repeat binding factor BBR participates in the transcriptional regulation of the homeobox gene *Bkn3*. Plant J.

[35] Kooiker M, Airoldi CA, Losa A, Manzotti PS, Finzi L, Kater MM, Colombo L (2005). BASIC PENTACYSTEINE1, a GA binding protein that induces conformational changes in the regulatory region of the homeotic *Arabidopsis* gene *SEEDSTICK*. Plant Cell.

[36] Berger N, Dubreucq B, Roudier F, Dubos C, Lepiniec L (2011). Transcriptional regulation of *Arabidopsis LEAFY COTYLEDON2* involves *RLE*, a *cis*-element that regulates trimethylation of histone H3 at Lysine-27. Plant Cell.

[37] Yu PH, Jiang N, Fu WM, Zheng GJ, Li GY, Feng BH, Chen TT, Ma JY, Li HB, Tao LX, Fu GF*.* ATP hydrolysis determines cold tolerance by regulating available energy for Glutathione synthesis in rice seedling plants. Rice (N.Y.). 2020;13(1):23. 10.1186/s12284-020-00383-7 PMC7145886 32274603

[38] Liu C, Yang XX, Yan ZS, Fan YJ, Feng GJ, Liu DJ (2019). Analysis of differential gene expression in cold-tolerant vs. cold-sensitive varieties of snap bean (*Phaseolus vulgaris* L.) in response to low temperature stress. Genes Genomics.

[39] Lee DH, Lee CB (2000). Chilling stress-induced changes of antioxidant enzymes in the leaves of cucumber: in gel enzyme activity assays. Plant Sci.

[40] Dong WK, Ma X, Jiang HY, Zhao CX, Ma HL (2020). Physiological and transcriptome analysis of Poa pratensis var anceps cv. Qinghai in response to cold stress. BMC Plant Biol.

[41] Zhang F, Ji SJ, Wei BD, Cheng SC, Wang YJ, Hao J, Wang SY, Zhou Q (2020). Transcriptome analysis of postharvest blueberries (*Vaccinium corymbosum* ’Duke’) in response to cold stress. BMC Plant Biol.

[42] Bartels D, Sunkar R (2005). Drought and salt tolerance in plants. Crit Rev Plant Sci.

[43] Feng XJ, Li SZ, Meng D, Di QH, Zhou MD, Yu XC, He CX, Yan Y, Wang J, Sun MT, Li YS (2023). *CsBPC2* is a key regulator of root growth and development. Physiol Plant.

[44] Zhu JH, Dong CH, Zhu J-K (2007). Interplay between cold-responsive gene regulation, metabolism and RNA processing during plant cold acclimation. Curr Opin Plant Biol.

[45] Agarwal M, Hao YJ, Kapoor A, Dong CH, Fujii H, Zheng XW, Zhu J-K (2006). A R2R3 type MYB transcription factor is involved in the cold regulation of CBF genes and in acquired freezing tolerance. J Biol Chem.

[46] Chinnusamy V, Zhu JH, Zhu J-K (2007). Cold stress regulation of gene expression in plants. Trends Plant Sci.

[47] Zhang X, Fowler SG, Cheng HM, Lou YG, Rhee SY, Stockinger EJ, Thomashow MF (2004). Freezing-sensitive tomato has a functional CBF cold response pathway, but a CBF regulon that differs from that of freezing-tolerant *Arabidopsis*. Plant Cell.

[48] Sanghera GS, Wani SH, Hussain W, Singh NB (2011). Engineering cold stress tolerance in crop plants. Curr Genomics.

[49] Kasuga M, Liu Q, Miura S, Yamaguchi-Shinozaki K, Shinozaki K (1999). Improving plant drought, salt, and freezing tolerance by gene transfer of a single stress-inducible transcription factor. Nat Biotechnol.

[50] Nakashima K, Yamaguchi-Shinozaki K (2006). Regulons involved in osmotic stress-responsive and cold stress-responsive gene expression in plants. Physiol Plantarum.

[51] Jaglo-Ottosen KR, Gilmour SJ, Zarka DG, Schabenberger O, Thomashow MF (1998). *Arabidopsis CBF1* overexpression induces *COR* genes and enhances freezing tolerance. Science.

[52] Maruyama K, Sakuma Y, Kasuga M, Ito Y, Seki M, Goda H, Shimada Y, Yoshida S, Shinozaki K, Yamaguchi-Shinozaki K (2004). Identification of cold-inducible downstream genes of the *Arabidopsis* DREB1A/CBF3 transcriptional factor using two microarray systems. Plant J.

[53] Okawa K, Nakayama K, Kakizaki T, Yamashita T, Inaba T (2008). Identification and characterization of Cor413im proteins as novel components of the chloroplast inner envelope. Plant Cell Environ.

[54] Jiang Y-P, Huang L-F, Cheng F, Zhou Y-H, Xia X-J, Mao W-H, Shi K, Yu J-Q (2013). Brassinosteroids accelerate recovery of photosynthetic apparatus from cold stress by balancing the electron partitioning, carboxylation and redox homeostasis in cucumber. Physiol Plant.

[55] Wang D-Z, Jin Y-N, Ding X-H, Wang W-J, Zhai S-S, Bai L-P, Guo Z-F (2017). Gene regulation and signal transduction in the ICE–CBF–COR signaling pathway during cold stress in plants. Biochemistr(Mosc).

[56] Eremina M, Unterholzner SJ, Rathnayake AI, Castellanos M, Khan M, Kugler KG, May ST, Mayer KF, Rozhon W, Poppenberger B (2016). Brassinosteroids participate in the control of basal and acquired freezing tolerance of plants. Proc Natl Acad Sci USA.

