# Supplementary material for: CsBPC2 is essential for cucumber survival under cold stress
Source: BMC Plant Biol. 2023 Nov 16;23:566. doi: 10.1186/s12870-023-04577-1 (PMC10652477; doi:10.1186/s12870-023-04577-1)
Supplement: Supplementary file 5 — Additional file 5. Figure S1. Heat map of common cold-responsive differential genes between WT and Csbpc2 mutants. A. Category one: the expression of the genes increased more in the WT than in mutants. B. Category two: the expression of the genes increased more in the mutants than in the WT. C. Category three: the expression of the genes decreased more in the mutants than in the WT. D. Category four: the expression of the genes decreased more in the WT than in mutants. Figure S2. Heat map of cold-responsive differential genes unique to WT. A. up-regulated genes; B. down-regulated genes. Figure S3. Heat map of cold-responsive differential genes unique to Csbpc2 mutants (L2,L3). A. up-regulated genes; B. down-regulated genes. Figure S4. Relative expression (2-ΔΔCt) of the differentially expressed genes. [file 12870_2023_4577_MOESM5_ESM.docx]

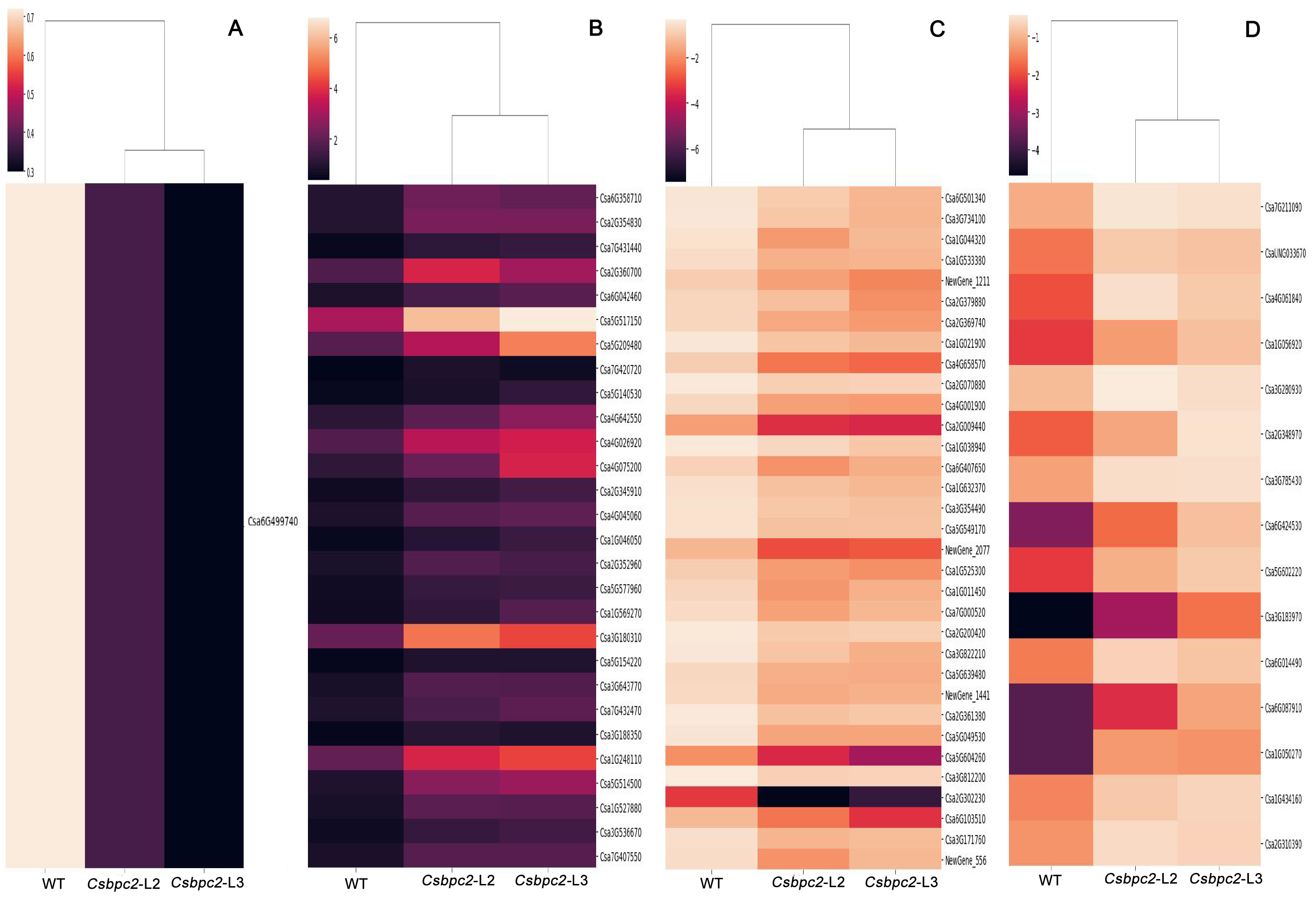


**Figure S1.** Heat map of common cold-responsive differential genes between WT and *Csbpc2* mutants. A.Category one: the expression of the genes increased more in the WT than in mutants. B. Category two: the expression of the genes increased more in the mutants than in the WT. C. Category three: the expression of the genes decreased more in the mutants than in the WT. D. Category four: the expression of the genes decreased more in the WT than in mutants


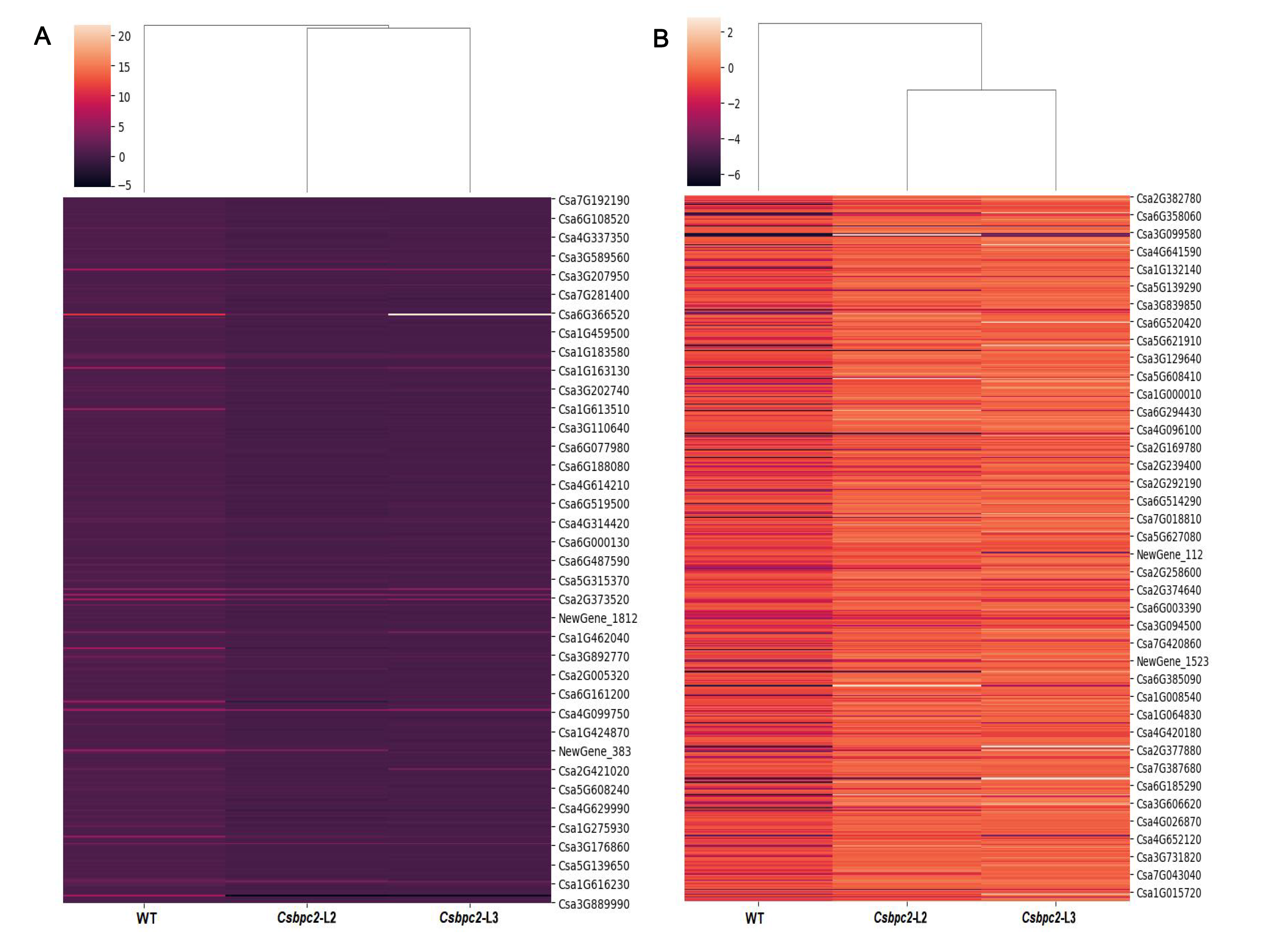


**Figure S2.** Heat map of cold-responsive differential genes unique to WT. A. up-regulated genes; B. down-regulated genes
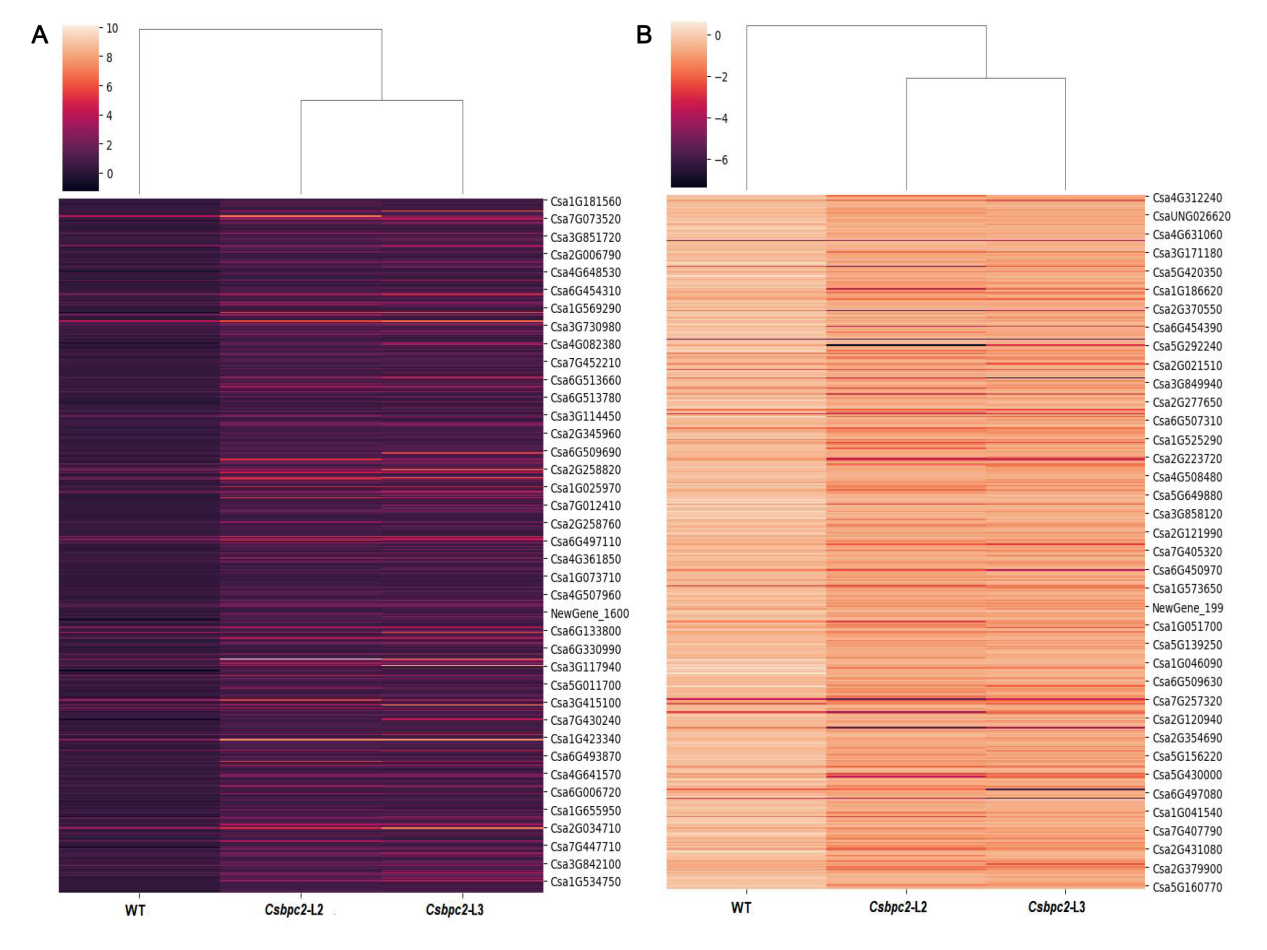


**Figure S3.** Heat map of cold-responsive differential genes unique to *Csbpc2* mutants (L2,L3). A. up-regulated genes; B. down-regulated genes


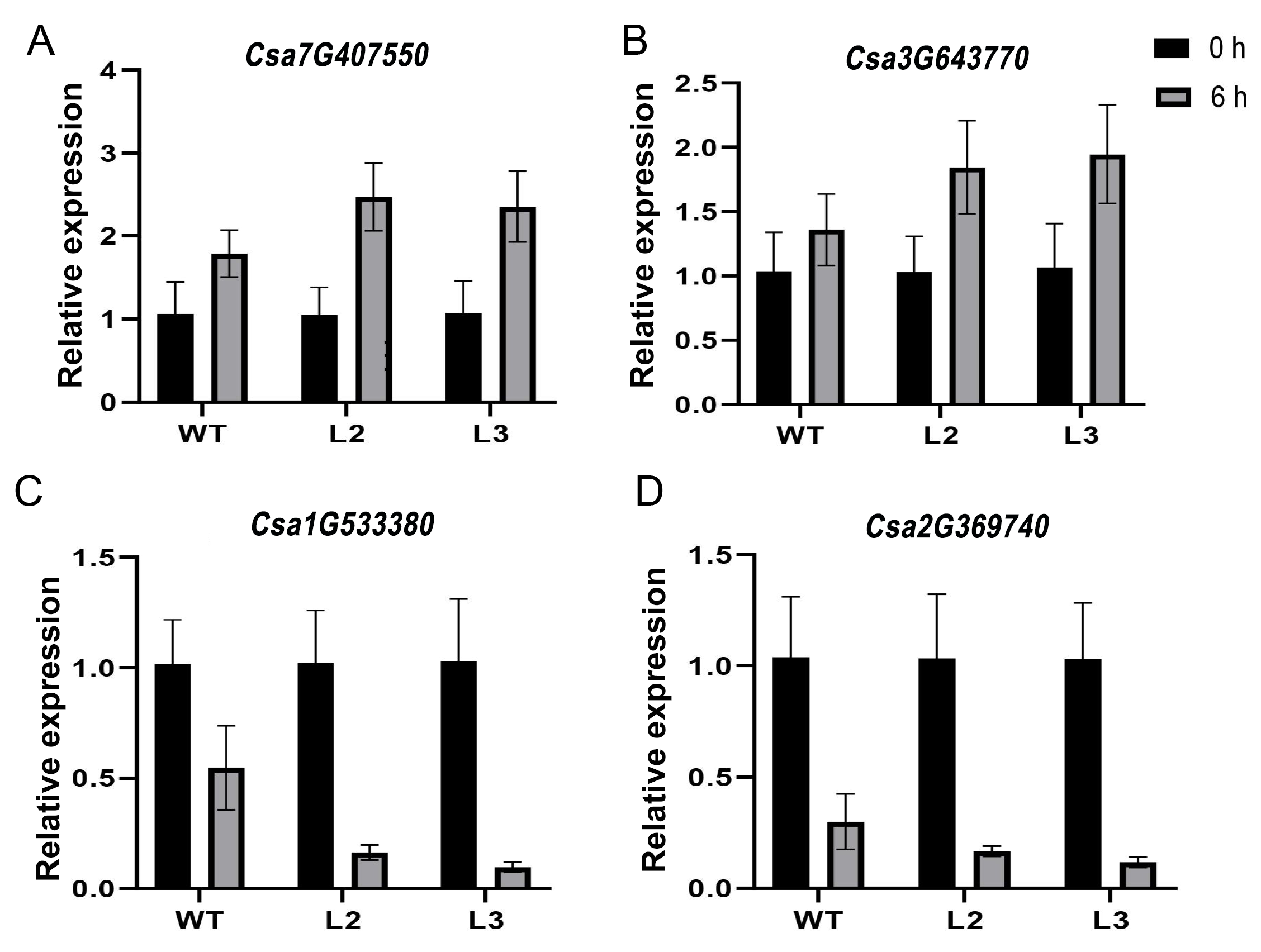


**Figure S4.** Relative expression (2^-ΔΔCt^) of the differentially expressed genes.
